# Heterogeneous atypical cell populations are present in blood of metastatic breast cancer patients

**DOI:** 10.1186/bcr3622

**Published:** 2014-03-06

**Authors:** Maryam B Lustberg, Priya Balasubramanian, Brandon Miller, Alejandra Garcia-Villa, Clayton Deighan, Yongqi Wu, Sarah Carothers, Michael Berger, Bhuvaneswari Ramaswamy, Erin R Macrae, Robert Wesolowski, Rachel M Layman, Ewa Mrozek, Xueliang Pan, Thomas A Summers, Charles L Shapiro, Jeffrey J Chalmers

**Affiliations:** 1 Stefanie Spielman Comprehensive Breast Center, Wexner Medical Center, The Ohio State University, 1145 Olentangy River Road, Columbus, OH 43212, USA; 2 William G Lowrie Department of Chemical and Biomolecular Engineering, The Ohio State University, 125A Koffolt Laboratories, 140 W 19th Ave, Columbus, OH 43210, USA; 3 Department of Pathology and Laboratory Services, Walter Reed National Military Medical Center, 8901 Rockville Pike, Bethesda, MD 20889, USA; 4 The Breast Cancer Research Program, The Ohio State University Comprehensive Cancer Center–Arthur G James Cancer Hospital and Solove Research Institute, 300 W 10th Avenue, Columbus, OH 43210, USA; 5 Center for Biostatistics, The Ohio State University, 2012 Kenny Road, Columbus, OH 43221, USA

## Abstract

**Introduction:**

Circulating tumor cells (CTCs) are commonly isolated from the blood by targeting the epithelial cell adhesion molecule (EpCAM) through positive selection. However, EpCAM can be downregulated during metastatic progression, or it can be initially not present. We designed the present prospective trial to characterize CTCs as well as other circulating cell populations in blood samples from women with metastatic breast cancer without EpCAM-dependent enrichment and/or isolation technology.

**Methods:**

A total of 32 patients with metastatic breast cancer were enrolled, and blood samples were processed using a previously described negative depletion immunomagnetic methodology. Samples from healthy volunteers were run as controls (*n* = 5). Multistep sequential labeling was performed to label and fix cell-surface markers followed by permeabilization for cytokeratins (CK) 8, 18 and 19. Multiparametric flow cytometry (FCM) analysis was conducted using a BD LSR II flow cytometer or a BD FACSAria II or FACSAria III cell sorter. Immunocytochemical staining on postenrichment specimens for DAPI, EpCAM, CD45, CK, epidermal growth factor receptor and vimentin was performed. Expression of these markers was visualized using confocal microscopy (CM).

**Results:**

CD45-negative/CK-positive (CD45− CK+) populations with EpCAM + and EpCAM − expression were identified with both FCM and CM from the negatively enriched patient samples. In addition, EpCAM + and EpCAM − populations that were CK + and coexpressing the pan-hematopoietic marker CD45 were also noted. There were more CK + EpCAM − events/ml than CK + EpCAM + events/ml in both the CD45− and CD45+ fractions (both statistically significant at *P* ≤ 0.0005). The number of CK + CD45− and CK + CD45+ events per milliliter in blood samples (regardless of EpCAM status) was higher in patient samples than in normal control samples (*P* ≤ 0.0005 and *P* ≤ 0.026, respectively). Further, a significant fraction of the CK + CD45+ events also expressed CD68, a marker associated with tumor-associated macrophages. Higher levels of CD45-CK + EpCAM − were associated with worse overall survival (*P* = 0.0292).

**Conclusions:**

Metastatic breast cancer patients have atypical cells that are CK + EpCAM − circulating in their blood. Because a substantial number of these patients do not have EpCAM + CTCs, additional studies are needed to evaluate the role of EpCAM − circulating cells as a prognostic and predictive marker.

## Introduction

A recently held multidisciplinary workshop titled “Lorentz Workshop Circulating Tumor Cell (CTC) Isolation and Diagnostics: Toward Routine Clinical Use” defined a CTC as a cell in the blood originating from either a primary or metastatic tumor and having properties that enable migration into the circulation [1]. It was also mentioned that a small subset of these CTCs may establish metastatic growth after seeding in a tissue niche, with some of them having undergone an epithelial–mesenchymal transition (EMT), in order to acquire the necessary mobility and invasiveness to promote metastasis [2]. Historically, the assumption that the CTC originated from an epithelial solid tumor facilitated isolation and identification technologies that would select for surface markers consistent with epithelial cells, such as the epithelial cell adhesion molecule (EpCAM) [3-6]. Beyond expressing EpCAM, the accepted definition of CTC requires that cells have the following (as determined by a trained operator): nuclei; cytokeratins (CKs) CK8, CK18 and CK19; no expression of the pan-hematopoietic marker CD45; and morphology consistent with a tumor cell. Based on these criteria, an elevated number of CTCs before treatment or after one cycle of treatment is an adverse prognostic factor in metastatic breast cancer [3-7]. Researchers in two phase III trials (SWOG-S0500, Southwest Oncology Group/National Cancer Institute, ClinicalTrials.gov ID NCT0032018 (USA); CirCe01, Institut Curie, ClinicalTrials.gov ID NCT01349842 (France)) currently underway are evaluating the use of EpCAM + CTCs as predictive markers of response to systemic therapy in metastatic breast cancer [8].

A significant challenge in using CTCs as a predictive marker is that more than one-third of patients with metastatic disease do not have detectable CTCs by EpCAM-based technology [9]. Patients with undetectable CTCs have a more favorable prognosis than patients with detectable CTCs. However, there may be individual patients with poor prognoses who have CTCs not captured by EpCAM-based positive enrichment, such as patients who have undergone EMT with downregulation of EpCAM and other epithelial markers. CTCs with an EMT-like phenotype have been reported recently by several groups [10-14]. In order to avoid the potential bias associated with positive selection methods, we have developed a negative enrichment strategy that relies on red blood cell (RBC) lysis followed by a combination of well-defined viscous flow and optimized magnetic forces to remove CD45-expressing leukocytes from patients’ whole-blood samples [15-17]. Using this enrichment system, we have identified CTCs in all breast cancer stages [12]. In studies conducted in patients with squamous cell carcinoma of the head and neck, we have shown significantly reduced disease-free survival with the presence of increased “putative” CTCs [11]. The term *putative* is used because EpCAM selection was not employed and, for those cells tested, EpCAM was not present. Furthermore, many CTCs expressed vimentin as well as epidermal growth factor receptor (EGFR), which is consistent with cells with a mesenchymal phenotype [10,18].

We hypothesized that (1) we could detect more CTCs and other atypical circulating cells if we used a non-EpCAM-based negative enrichment approach and (2) using flow cytometry (FCM) and confocal immunocytochemistry (ICC) after negative depletion would allow these putative CTCs to be phenotyped, further assisting in the classification. To test this hypothesis, peripheral blood samples from patients with metastatic breast cancer were obtained, subjected to the negative depletion process and examined by multiparametric FCM and ICC analysis to characterize the different subpopulations of EpCAM + and EpCAM − cells.

## Methods

### Cell cultures

The breast cancer cell lines MCF-7 (HTB-22) and MDA-MB-231 (HTB-26) were procured from the American Type Culture Collection (ATCC; Manassas, VA, USA) and grown to mid-log phase in ATCC-specified culture media at 37°C in a 5% CO_2_ atmosphere when specified. Cells were harvested by washing the adherent cells with phosphate-buffered saline and then incubating them with Accutase (AT104; Innovative Cell Technologies, San Diego, CA, USA) for 5 minutes at 37°C to remove the attached cells from the culture flask. Accutase was then neutralized with the culture media before the cells were pelleted at 350 × *g* for 5 minutes. Cells were resuspended in appropriate medium, depending on the downstream application.

### Patient samples and blood collection

Thirty-two metastatic breast cancer patients, who were older than 18 years of age and had two or less prior lines of systemic therapy were enrolled in The Ohio State University Cancer Institutional Review Board (IRB)–approved protocol. All patients gave their informed consent to participate in the study. Blood samples were collected after several standard blood tubes were drawn for routine chemotherapy laboratory prior to initiation of a new line of systemic therapy. Peripheral blood (7.8 to 17.7 ml) was collected in BD Vacutainer tubes (366643; BD Biosciences, San Jose, CA, USA) for CTC enumeration and processed within 4 hours of blood collection.

### Normal control blood collection

Blood was collected from healthy volunteer donors (*n* = 5) after obtaining their informed consent using an IRB-approved protocol at The Ohio State University (OSU) Medical Center and processed in the same manner as the patient samples. In addition, 11 to 18 ml of peripheral blood was collected from ten individuals without a known diagnosis of breast cancer before they underwent diagnostic procedures at the Walter Reed Army Medical Center Comprehensive Breast Care Center. Their blood samples were utilized for ICC as confocal control specimens but not were used for FCM. The protocol was approved by the Cancer Institutional Review Board of the Walter Reed Army Medical Center (IRBnet 354344), initiated in January 2011, and informed, written consent was obtained from all healthy volunteer donors. Peripheral venous blood was collected in BD Vacutainer tubes (362753; BD Biosciences) and shipped overnight to OSU for immediate processing. All samples were processed within 30 hours of blood draw.

### Sample processing for negative depletion enrichment

**Figure 1:**
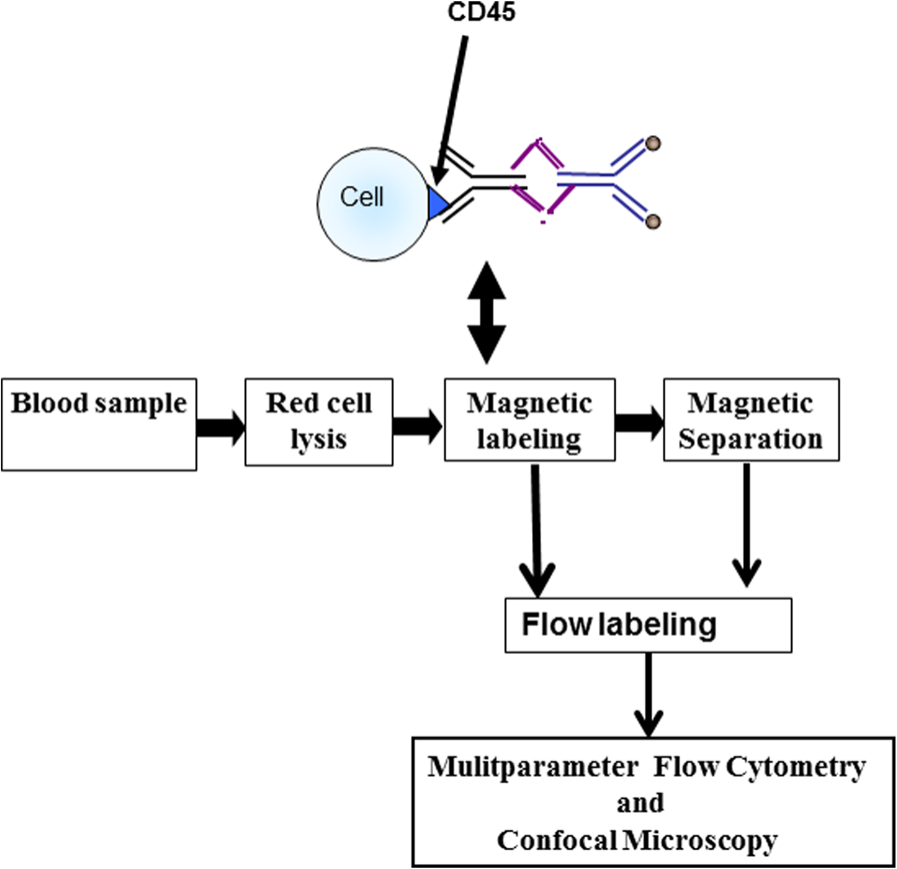
Enrichment and analysis methodology used in this study.

Blood samples were kept at ambient temperature and processed using a negative depletion immunomagnetic methodology described previously [15,16,18]. Briefly, blood samples were subjected to a RBC lysis step and labeled with anti-CD45 tetrameric antibody complex (STEMCELL Technologies, Vancouver, BC, Canada). Magnetic nanoparticles were added and incubated with the cell suspension, which was run through the quadrupole magnetic sorter system (Figure 1). A measure of the effectiveness of the enrichment of the samples is represented as the log_10_ of the ratio of the total number of nucleated cells before processing to the total number of nucleated cells after processing [15,19].

### Multiparameter flow cytometry

Sample aliquots were taken prior to and after magnetic labeling for setting up gating controls for multiparametric FCM. The enriched sample after separation was split into two fractions. One fraction was stained using a multistep sequential labeling protocol for FCM. Briefly, the FCM sample was labeled for surface markers, fixed with 4% paraformaldehyde to stabilize the surface staining, permeabilized with 0.1% Triton X-100 and stained for intracellular proteins. The antibody fluorescent probe combination used can be found in Additional file 1: Table S1. A BD LSR II flow cytometer or a BD FACSAria II cell sorter (BD Biosciences) equipped with three excitation lasers (405 nm, 488 nm and 633 nm) was used for initial FCM analysis. Both automatic and manual compensation were evaluated for each patient sample.

During the study period, a BD FACSAria III cell sorter was acquired. The FACSAria III is equipped with 355-, 488-, 561- and 635-nm lasers, which allows for fluorescein isothiocyanate and phycoerythrin dyes to be excited with different lasers (488 nm and 561 nm) incident to the cell at different locations in the stream, thereby significantly reducing the need for compensation.

To determine whether the exclusion of nonviable cells, determined with the LIVE/DEAD Fixable Aqua Dead Cell Stain Kit (L34957; Life Technologies, Carlsbad, CA, USA), would change the staining distribution of cells, selected patient samples for this assay were assessed using the FACSAria III system. All gates were determined on the basis of unstained and single-color controls of each patient sample, control samples from healthy volunteers or buffy coats. Because at most two colors were excited by a single laser (one dye per laser on the FACSAria III), this gating strategy was equivalent to the fluorescence minus one approach. For the samples using the viability assay, the initial step included using a side scatter width setting to exclude obvious doublets.

### Confocal immunocytochemistry analysis of negatively enriched patient samples

After negative depletion, an aliquot of cell suspension preserved in 10% neutral buffered formalin was retained for ICC staining and confocal microscopy (CM) analysis. The antibody-fluorescent probe combination used can be found in the Additional file 2: Supplemental Information. To facilitate high-quality image acquisition, an Olympus FluoView FV1000 laser scanning confocal microscope (Olympus Imaging America, Center Valley, PA, USA) equipped with 405-nm, 488-nm, 543-nm and 633-nm lasers was used. All images were acquired, and processed, using the Olympus FluoView Ver3.0 software (Olympus America), which records all relevant instrument settings and automatically determines pixel size (size scales in the *x*, *y* and *z* directions). Additional file 2: Supplemental Information contains images that indicate the excitation filters or laser used, the emission filters used and the excitation and emission spectra of the dyes.

### Statistical analysis

Negatively enriched specimens were further classified into subpopulations according to the expression levels of CD45, CK and EpCAM, as visualized by FCM. CK + events per milliliter of blood sample volume in each subpopulation were summarized. One sample sign test was used to compare the differences between any two subpopulations from patient samples. A Wilcoxon rank-sum test (Mann–Whitney *U* tests) was used to test the differences between patient and healthy volunteer control groups. Sensitivity analyses were conducted to confirm the conclusions using a paired *t*-test (two-sample *t*-test with unequal variance after proper data transformation). The *P*-values without multiple comparison adjustment are reported.

### Kaplan–Meier plots

Kaplan–Meier (KM analysis) was performed on several of the subpopulations identified by multiparametric FCM. Given the distinct grouping of cohorts required for the KM estimator, we chose to use binary grouping: patients with number of events per milliliter of blood sample of the specific combination of markers below a cutoff and above the cutoff. This cutoff was initially set at one standard deviation above the mean of normal blood controls, which works well when the specific cell population is not rare. However, as is generally recognized, the detection and analysis of rare events (approximately 0.1% and less), such as CTCs in peripheral blood specimens by FCM, is challenging.

Authors of several experimental and theoretical reports have attempted to provide a statistically based approach to reliably enumerate rare events by FCM. One such approach is the use of Poisson distribution statistics, which deal with the probability distribution of rare events. A Poisson coefficient of variation (CV) associated with rare event data obtained in a flow cytometer can be estimated by the following equation:

(1) $$\mathrm{CV}=\frac{100}{\sqrt{r}}$$

where, *r* is the number of events detected [20-23]. Equation (1) indicates that a CV of 10% requires 100 events to be positively detected and that a CV of 5% requires 400 events.

The smallest blood sample we obtained in this study was approximately 4 ml. To achieve a CV of 5%, 400 positive events needed to be detected. Therefore, in this study, to be able to associate a CV of 5% to a measurement, at least 100 events/ml of blood sample volume were needed. Consequently, when the mean plus one standard deviation of the normal controls was less than 100 events/ml of blood sample volume, we set 100 events/ml of blood sample volume as a cutoff. When the mean plus standard deviation of the normal controls was greater than 100, we set this value as the cutoff.

## Results

### Clinical characteristics

Thirty-two patients with metastatic breast cancer were enrolled, including fifteen triple-negative (TNBC) estrogen receptor, progesterone receptor and Her2 (ER − PR − Her2−), fourteen ER + Her2− and three Her2+ patients. The median age of the patients was 55 years (range, 35 to 75 years). All patients had visceral involvement in one or more sites. The patients’ clinical characteristics are summarized in Table 1.

**Table 1:** **Clinical characteristics of the patients**^**a**^

**Characteristics**	**All patients (*N*** **= 32)**
Age, years	
Median	55
Range	35 to 75
Race	
White	30 (94)
African American	2 (6)
ECOG performance status grade	
0	8 (25)
1	20 (62)
2	4 (13)
Menopausal status	
Premenopausal	4 (12)
Postmenopausal	28 (88)
Number of metastatic sites	
1	8 (25)
2	12 (38)
≥3	12 (38)
Sites of metastasis	
Bone	20 (62)
Lung	10 (31)
Liver	15 (47)
Brain	8 (25)
Type of breast cancer	
ER − PR − Her2− (TNBC)	15 (47)
ER + Her2 − (ER+)	14 (44)
Her2+	3 (9)

^a^ECOG, Eastern Cooperative Oncology Group; ER, Estrogen receptor; PR, Progesterone receptor; TNBC, Triple-negative breast cancer.

### Immunomagnetic negative depletion of patient samples

Nucleated cell counts were determined prior to the RBC lysis step, after RBC lysis and after magnetic depletion of CD45 + -labeled cells. The average recovery of nucleated cells after RBC lysis was 58%, the average nucleated log_10_ depletion was 2.7 and the total log_10_ depletion was 5.6 (range, 4.0 to 6.2). (A log_10_ depletion of 6.0 corresponds to only one of one million cells left after processing).

### Flow cytometry analysis of negatively enriched blood

**Figure 2:**
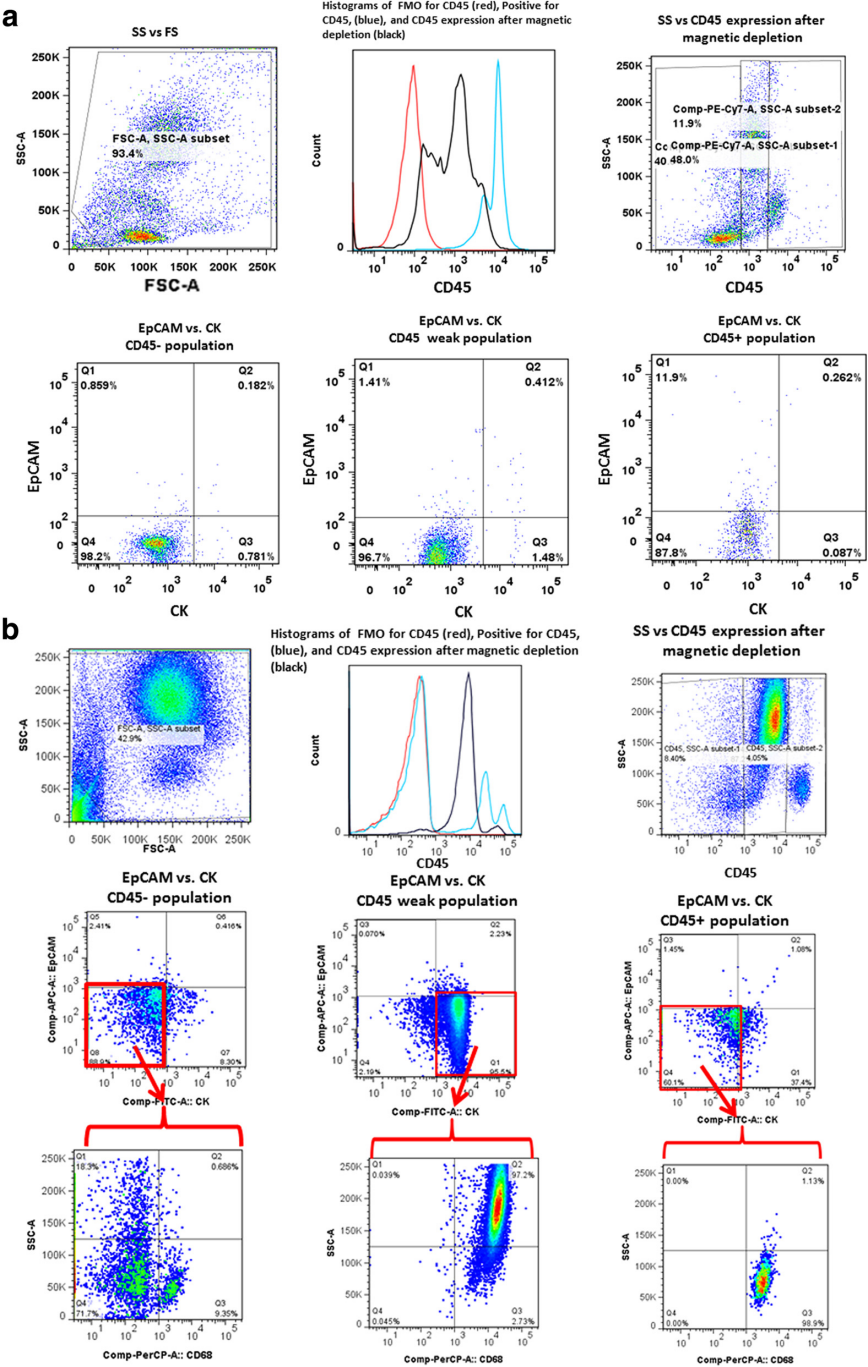
**Comparative flow cytometry analyses. (a)** CD45-depleted, buffy coat blood sample. **(b)** Representative triple-negative breast cancer patient blood sample. All samples were labeled with antibodies against CD45; cytokeratins (CKs) CK8, CK18 and CK19; and epithelial cell adhesion molecule (EpCAM). (a) and (b) are progression of plots. Top row, left to right: forward scatter area (FSC-A) and side scatter area (SSC-A); histograms of unlabeled (red), CD45-stained (blue) and population expression after magnetic depletion (black); and dot plot of SSC versus CD45 expression gated into CD45− expression, low CD45 expression and high CD45 high expression. Bottom row, left to right dot plots of EpCAM expression versus CK expression for the CD45−, CD45 low and CD45 high populations. In part (b), a third row of plots is presented in which the events in the red outlined quadrant in the second row are further investigated with respect to SS verses CD68 expression. APC, Allophycocyanin; Cy7, Cyanine 7; FITC, Fluorescein isothiocyanate; FMO, Fluorescence minus one; PE, Phycoerythrin; PerCP, Peridinin chlorophyll protein.

Blood samples were analyzed by FCM after enrichment. Data from spiking studies of cell lines as well as isotype control studies are shown in Additional file 2: Supplemental Data. Figure 2a illustrates the CD45 gating strategy for negative enriched control (healthy volunteer), and Figure 2b shows the analysis of a representative sample from a patient with metastatic breast cancer. Four different CK + subpopulations were identified by FCM using the antibody conjugates targeting CD45, CK and EpCAM. Selected samples obtained using the FACSAria III were also stained with the LIVE/DEAD Fixable Aqua Dead Cell Stain Kit (*n* = 5). No significant difference in the ratio of the four different subfractions was observed when dead cells were excluded compared to when they were not excluded (data not shown).

As shown in Figure 2b, and consistent with all of the CD45 + CK + cells observed in all of the FCM analyses, the vast majority of the cells weakly expressed CD45 (within an order of magnitude of signal intensity used to “gate” between positive and negative cells) and appeared in the side scatter and forward scatter plots in the region consistent with more granulocyte-type cells. This observation is in contrast to more typically “bright” CD45 cells, which demonstrate less granulocytic characteristics and are presumably more lymphocyte-like and are removed in the magnetic depletion step.

### Additional characterization of the CD45 + CK + events in flow cytometry analysis

To begin to further characterize this CD45 + CK + subpopulation, we added an anti-CD68 antibody added to the staining protocol for 11 patient samples. For the samples tested, more than 90% of the CD45 + CK + events were CD68-positive. One representative sample is presented in the bottom row of Figure 2b. Each dot plot in the bottom row corresponds to the specific quadrants in the middle row highlighted in red. The CD45 + CK + EPCAM − population (middle row, center dot plot and red-highlighted quadrant) is clearly positive for CD68. The location of CD68+ cells on the side scatter axis is consistent with the location of larger granular cells, such as monocyte- and/or macrophage-like cells. Although there are other weakly positive CD68 populations in the two other dot plots in the third row, the strongest CD68 positivity is present only in the CD45 + CK + EpCAM − population.

### Distinct subpopulations of atypical cells are present in metastatic breast cancer

**Figure 3:**
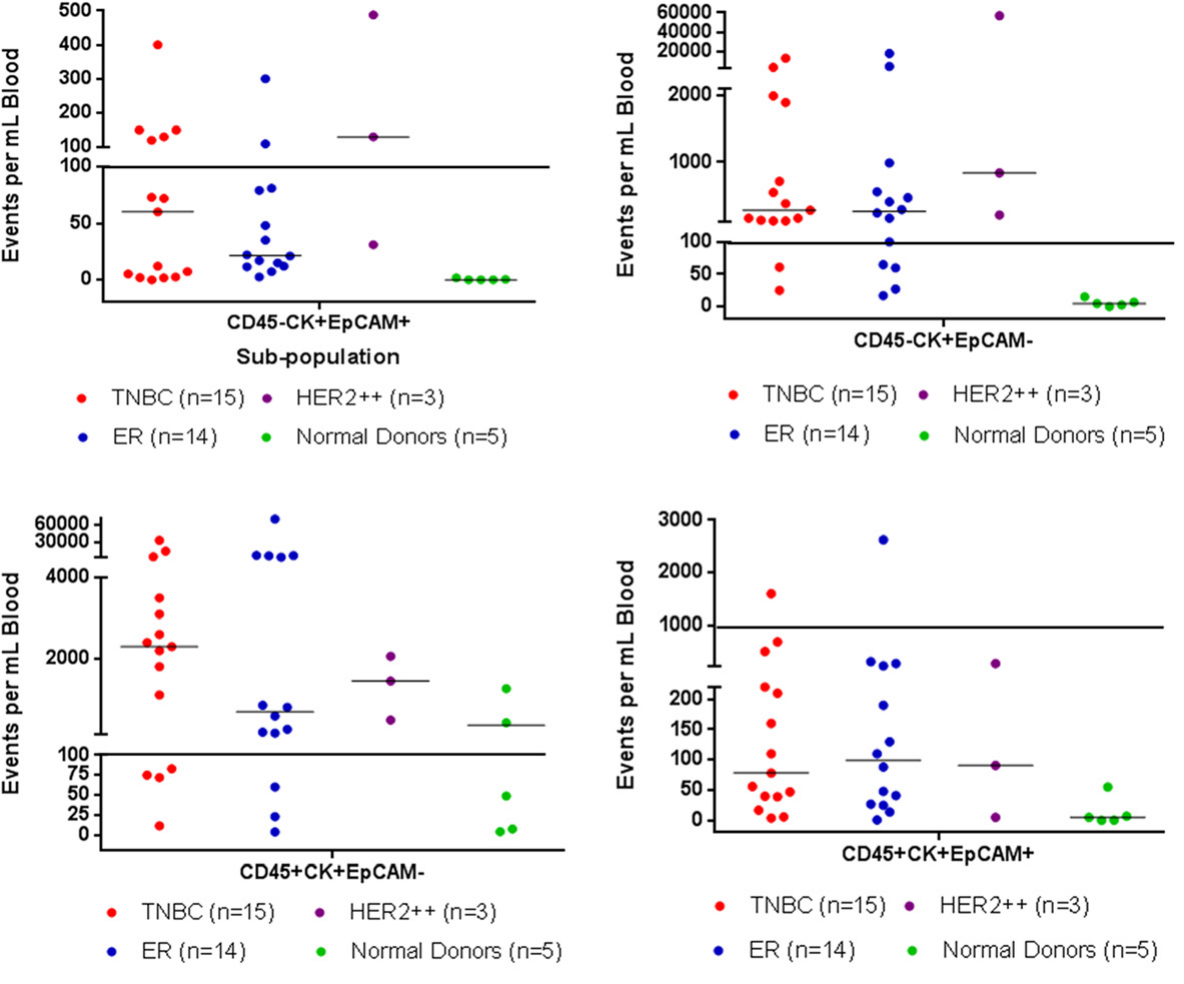
**Scatterplots of the estimated number of cytokeratin-positive events per milliliter of blood sample volume as determined by flow cytometry.** The solid black lines correspond to a Poisson distribution coefficient of variation of 5%. Note that two normal donor specimens have more than 100 CD45 + CK + EpCAM − events per milliliter of blood sample volume. CK, Cytokeratin; EpCAM, Epithelial cell adhesion molecule; ER, Estrogen receptor; TNBC, Triple-negative breast cancer.

Plots of the different subpopulations of the enriched patient samples in terms of the number of events per milliliter of blood sample volume, as identified by FCM, are presented in Figure 3 (number of CD45− and CD45+ events per milliliter of blood sample volume). For comparison, the same subpopulations are also presented for the healthy donors. As shown in Table 2, the CD45 + CK + EpCAM−, CD45 + CK + EpCAM+, CD45 − CK + EpCAM − and CD45 − CK + EpCAM + events per milliliter of blood sample volume in patient samples were all significantly higher than those in healthy control samples (*P* = 0.03, *P* = 0.0007, *P* = 0.0004 and *P* = 0.0008, respectively). The numbers of CK + CD45− and CK + CD45+ events per milliliter of blood sample volume, regardless of EpCAM status, were also significantly higher in patient samples than in control samples (*P* ≤ 0.0005 and *P* ≤ 0.026, respectively). Consistent with the generally accepted concept that the concentration of the traditionally defined CTCs is not high enough to be routinely detected in FCM analysis without sampling large blood volumes (that is, greater than 20 ml), the CD45 − CK + EpCAM + population concentrations were significantly greater than the control; however, most of the concentrations were below the 100 events per milliliter of blood sample volume threshold, which corresponded to a CV of 5% (black solid lines in Figure 3). The only subpopulation where the mean plus one standard deviation of events in the controls was greater than 100 events per milliliter of blood sample volume was CD45 + CK + EpCAM−. Consequently, for this subpopulation, the threshold was set as the mean plus one standard deviation of the controls for that subpopulation (1,000 events per milliliter of blood sample volume).

**Table 2:** **Atypical circulating subpopulations**^**a**^

**Subpopulations**	**CD45-CK + EpCAM+**	**CD45-CK + EpCAM−**	**CD45** + **CK + EpCAM+**	**CD45 + CK + EpCAM−**
Patient samples (*n* = 32)	33 (10.5 to 112.5)	275 (110 to 877.5)	89.5 (36 to 224.5)	1,623 (163.5 to 3,725)
Control (*n* = 5)	0 (0 to 0.4)	4.6 (2.3 to 6.7)	5 (0.5 to 7.1)	48.9 (7.4 to 402.1)
*P*-values	0.0008	0.0004	0.0007	0.03

^a^CK, Cytokeratin; EpCAM, Epithelial cell adhesion molecule. The values in each cell are medians (25% to 75% quartile range). *P*-values were calculated using the Mann–Whitney *U* tests of the subpopulations between control and patient samples.

Additional comparisons of the subpopulations present in patient samples highlights that there were significantly more CK + EpCAM − events than CK + EpCAM + events in both CD45− and CD45+ fractions (both *P* < 0.0005). There were no significant differences among the breast cancer subtypes (Figure 3).

### Clinical outcome correlations

**Figure 4:**
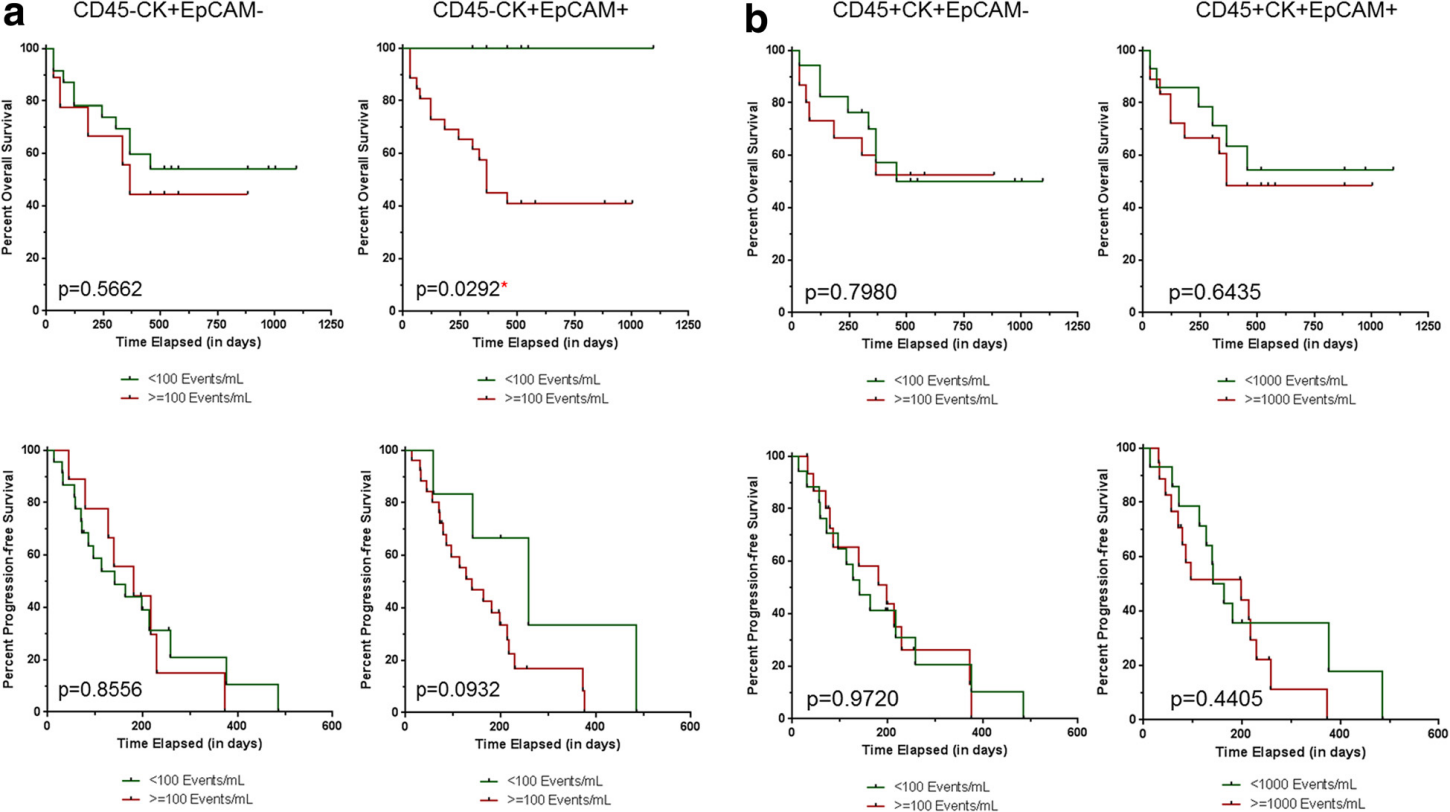
**Kaplan–Meier plots of progression-free survival and overall survival of 15 triple-negative breast cancer patients combined with 17 estrogen receptor–positive or Her2-positive patients. (a)** Plots for CD45-CK + EpCAM+/EpCAM − subpopulations are shown. **(b)** Plots for CD45 + CK + EpCAM+/EpCAM − subpopulations are shown. CK, Cytokeratin; EpCAM, Epithelial cell adhesion molecule.

Combining all three types of metastatic breast cancer, we asked whether elevated concentrations of these various subtypes would be predictive of progression-free survival or overall survival (OS) as determined by KM estimator analysis. As a binary cutoff for the KM analysis, we used the criteria for elevation that the concentration of events per milliliter of blood sample volume should have a CV of at least 5% and 100 or more events per milliliter of blood sample volume for the CD45 − CK + EpCAM − population. For the CD45 + CK + EpCAM − population, we used the mean plus one standard deviation of the normal patient population as a cutoff. These four KM plots are presented in Figure 4. Total events of 100 or more per milliliter of blood sample volume were associated with significantly poorer OS (*P* = 0.029).

### Confirmation of flow cytometry–defined subpopulations using immunocytochemistry confocal microscopy

**Figure 5:**
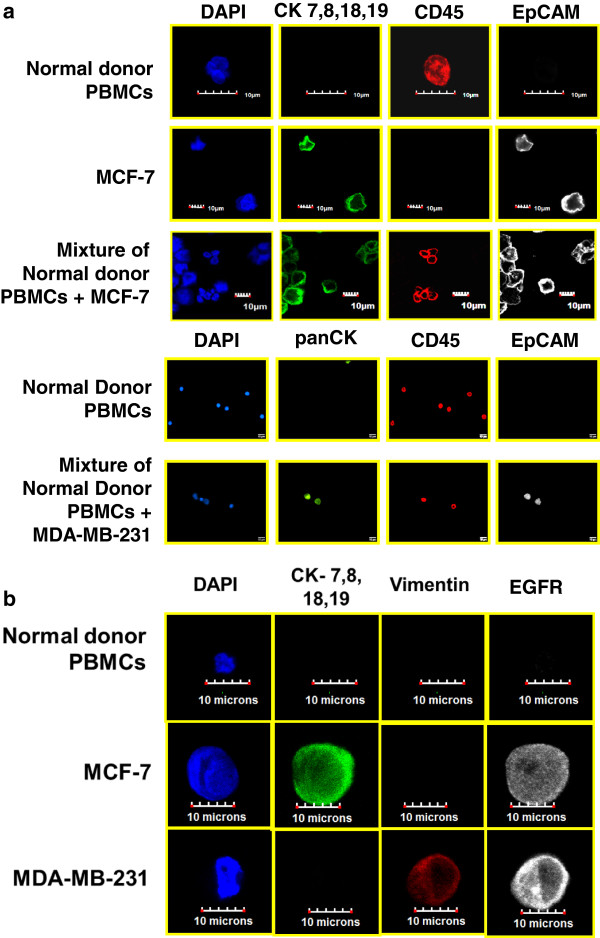
**Staining controls for different phenotypic markers used. (a)** The top three rows are confocal immunofluorescent images of normal donor peripheral blood mononuclear cells (PBMCs), MCF-7 and a mixture of normal donor PBMCs and MCF-7 stained with cytokeratin (CK) antibody (green), CD45 antibody (red), epithelial cell adhesion molecule (EpCAM) (white) and 4′,6-diamidino-2-phenylindole (DAPI; blue). The bottom two rows of images show healthy donor PBMCs and MDA-MB-231 stained with the same antibody dye conjugates and the CK antibody replaced by pan-CK versions. **(b)** Confocal immunofluorescent images show healthy donor PBMCs, MCF-7 and MDA-MB-231 stained with DAPI (blue); anti-CK7, anti-CK8, anti-CK18 and anti-CK19 antibody (green); antivimentin (red); and anti–epidermal growth factor receptor (EGFR; white).

To provide independent visual confirmation of the CD45− and CD45+ subtypes characterized by FCM analysis, four-color epifluorescence and confocal analysis of cytospins from among several of the enriched metastatic samples was conducted. Figures 5a and 5b show various combinations of the antibody fluoroprobes used in this study, confirming the specificity of Alexa Fluor custom-conjugated antibodies (Molecular Probes, Sunnyvale, CA, USA) to two breast cancer cell lines (MCF-7 and MDA-MB-231) and normal donor peripheral blood mononuclear cells. Note that MCF-7 is positive for all except CD45 and vimentin and MDA-MB-231 is negative for CK8, CK18 and CK19 but positive for at least one of CK1, CK4, CK5, CK6, CK10 or CK13 using a pan-CK antibody (Sigma-Aldrich, St Louis, MO, USA). Additional file 2: Supplemental Information provides further examples of controls, both using epifluorescence and CM.

**Figure 6:**
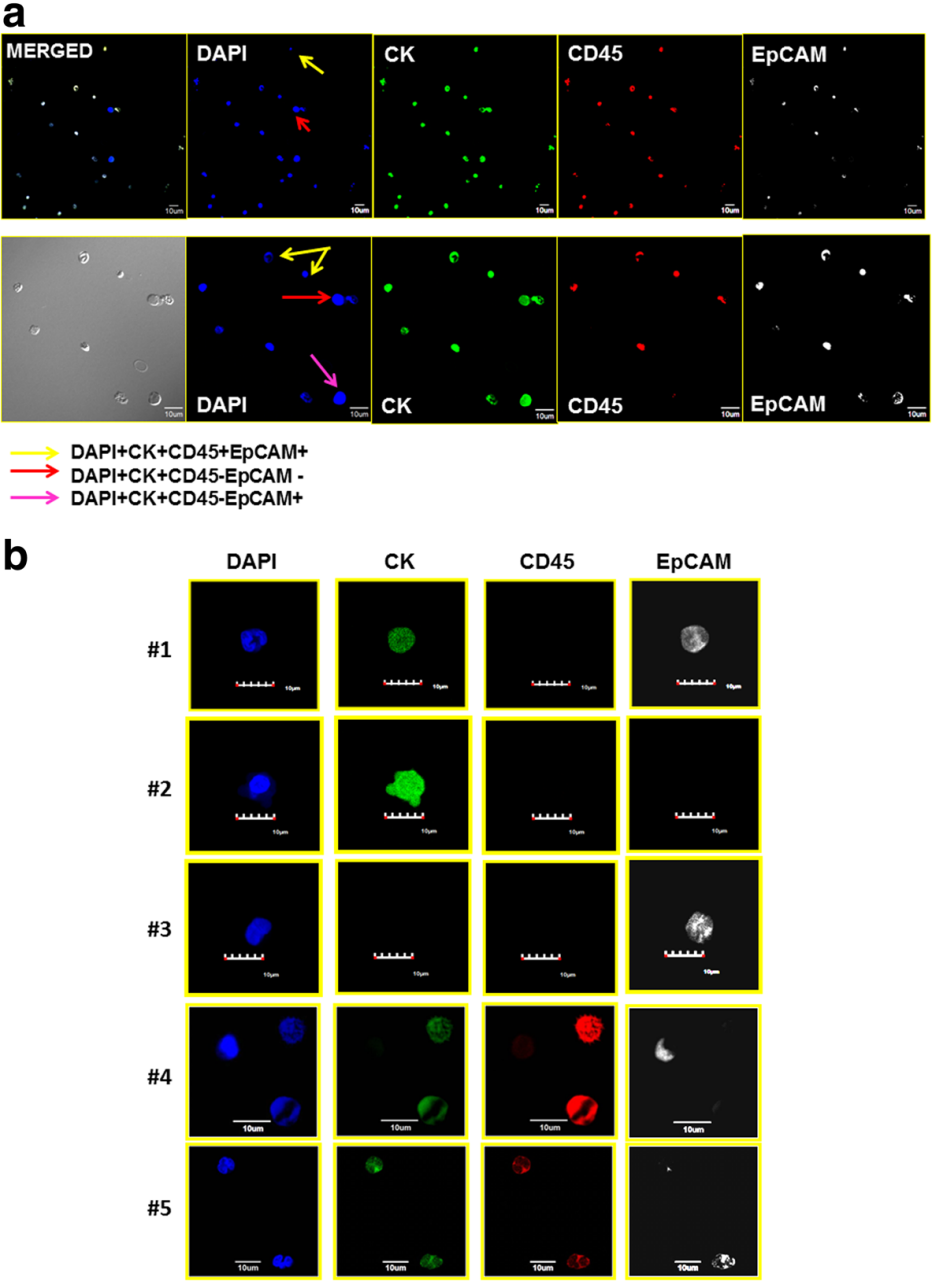
**Examples of different cell types found in the peripheral blood of triple-negative breast cancer patients. (a)** Peripheral blood mononuclear cells (PBMCs) of representative triple-negative breast cancer patients shown in low- and high-magnification confocal microscopy immunofluorescent images stained with cytokeratin (CK) antibody (green), CD45 antibody (red), epithelial cell adhesion molecule (EpCAM) (white) and 4′,6-diamidino-2-phenylindole (DAPI; blue). To assist in the identification of different cell types, based on staining pattern, we added arrows of different color according to the key provided. **(b)** High-magnification confocal images from a patient peripheral blood sample highlighting the different staining combinations observed.

Figure 6a presents low-magnification and high-magnification confocal images of a representative patient (whose FCM data are presented in Figure 2b) confirming the presence of various positive and negative combinations of CK and EpCAM staining on both CD45− and CD45+ subpopulations visualized by FCM. Colored arrows are used in Figure 6 to facilitate identification of several of the subtypes of interest in this study. It should be noted that it is very rare to find a DAPI + CD45 + CK-EpCAM − cell in these enriched samples, given the average log_10_ value of 2.7 for depletion of nucleated cells. Additional file 2: Supplemental Information provides low-magnification epifluorescent images of a patient sample prior to and after magnetic depletion.

Figure 6b presents high-magnification representative confocal images of expression of a traditional CTC (row 1), a putative CTC that is EpCAM − (row 2), a cell with absent CKs (CK8, CK18 and CK19) but EpCAM + (row 3), an atypical cell that is EpCAM + but absent CKs (CK8, CK18 and CK19) and series of CD45 + CK + cells that were recorded in almost all of the FCM analyses (rows 4 and 5).

### Multiparametric immunocytochemistry with vimentin and epidermal growth factor receptor staining

**Figure 7:**
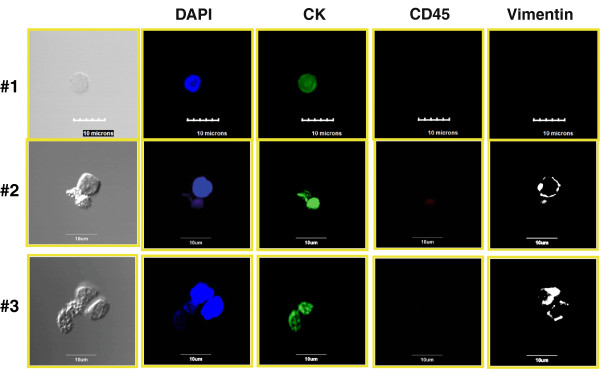
**Confocal immunofluorescent images of four representative triple-negative breast cancer patients.** Images are stained with cytokeratin (CK) antibody (green), CD45 antibody (red), vimentin (white) and 4′,6-diamidino-2-phenylindole (DAPI; blue). Note that the vimentin-positive cells are negative for the hematopoietic marker CD45. DIC, Differential interference contrast images; EGFR, Epidermal growth factor receptor.

**Figure 8:**
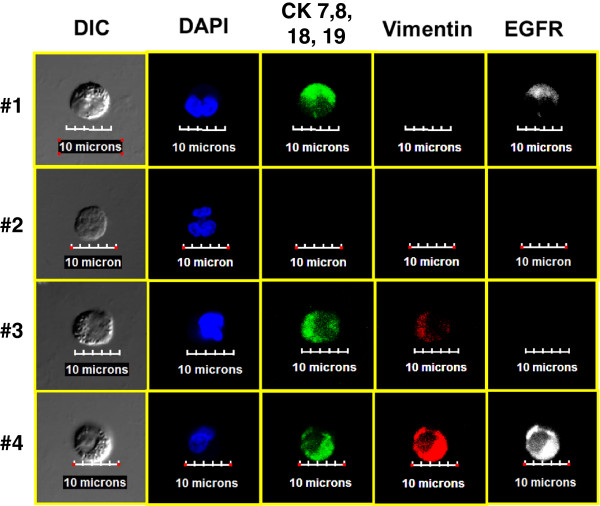
**Further combination of confocal images.** Images were produced by cytospin of a triple-negative breast cancer blood sample stained with 4′,6-diamidino-2-phenylindole (DAPI); antibodies against cytokeratins CK7, CK8, CK18 and CK19; anti-vimentin; and anti–epidermal growth factor receptor (EGFR). The four rows represent the four different combinations observed in the blood sample from this patient.

The FCM analysis and the confocal images indicate that other rare cells that do not fit the traditional definition of CTCs were present (that is, CD45 − CK + EpCAM−). To further phenotypically characterize these cells, different staining protocols were used, including replacing EpCAM and/or CD45 with vimentin or EGFR. Figure 7 presents a cytospin of enriched TNBC patient blood stained with 4′,6-diamidino-2-phenylindole (DAPI), CD45, CK and vimentin replacing EpCAM. Row 1 shows a traditional CTC negative for vimentin. Rows 2 and 3 show CD45− cells that have both epithelial (CK) and mesenchymal (vimentin) marker expression. Figure 8 presents further analysis in which EGFR replaces the CD45 staining. The four rows represent the four different staining combinations observed in the same cytospin of the blood sample from the same patient, again demonstrating subpopulation heterogeneity in metastatic patients. It should also be noted that CD45 was used in the examples presented in Figure 7 and that all of the vimentin-positive cells are CD45− and therefore not traditionally defined hematopoietic cells. In Figure 8, though CD45 was not used, the vimentin-positive cells are positive for either EGFR or cytokeratins, further decreasing the probability that these cells are of hematopoietic origin as sometimes suggested.

## Discussion

The currently accepted definition of a CTC is a nucleated cell that is positive for epithelial marker expression of EpCAM and cytokeratins CK8, CK18 and CK19 and negative for the pan-hematopoietic marker CD45. In addition to identifying these traditional CTCs, we show herein that there are other abnormal circulating cell populations present in blood samples from metastatic breast cancer patients. These populations include EpCAM − cells that are CK+, cells that express mesenchymal markers but few or no epithelial markers and cells which express both hematopoietic and epithelial makers. These findings highlight the fact that blood from metastatic breast cancer patients has a heterogeneous mixture of abnormal cells and is consistent with other reports of heterogeneous mixtures of putative CTCs [4,13,14,24].

Multiple studies have shown that CTCs detected by EpCAM + selection are prognostic in metastatic breast cancer [3,9,25-29]. Current ongoing studies are evaluating how and if these classical CTCs can be utilized as predictive markers in treatment decision-making [8]. However, on average, only up to 65% of patients with metastatic breast cancer have identifiable CTCs that fit these criteria [3,9]. There is wide speculation regarding why patients with metastatic breast cancer may have undetectable CTCs. As a group, patients with undetectable CTCs may have a better prognosis, but the lack of circulating biomarkers still can make evaluation of targeted therapies challenging. Additionally, it is possible that some tumors with aggressive features may have undergone EMT, which would downregulate epithelial markers such as EpCAM. Further, the molecularly classified basal-like breast tumors have a low or nonexistent expression of EpCAM and have increased expression of mesenchymal markers [30]. Studies utilizing cell lines have established the limitation of EpCAM + selection in recovering basal-like tumor cell lines [31], although no definitive study has clearly illustrated that this type of enrichment is not successful in basaloid tumors. Moreover, several studies have shown the EpCAM + selection is still a reliable prognostic marker in patients with metastatic TNBC [32-35].

However, the question remains whether there are additional cell populations of interest that could be missed using EpCAM preselection in patients with metastatic breast cancer. Konigsberg *et al*. compared two CTC isolation methods: an immunomagnetic positive selection approach targeting EpCAM (MACS CD326 (human epithelial antigen) MicroBeads; Miltenyi Biotec, San Diego, CA, USA) and an antibody-independent, density gradient centrifugation device which assumes CTCs have distinct, different densities compared to normal blood components (OncoQuick Plus; Greiner Bio-One, Frickenhausen, Germany) [36]. Their spiking studies confirmed the limitations of relying on a positive selection targeting EpCAM to identify all atypical cell populations within a given sample across a heterogeneous disease, consistent with other cell line studies [31]. However, it should be noted that their study included only CK markers and did not further characterize these cells. In addition, studies demonstrating the clinical utility of OncoQuick Plus isolated CTCs are limited. The clinical significance of EpCAM − CTC is not clear. Most recently, EpCAM − CTCs isolated by FCM and having a Notch1+/EGFR+/Her2+/EpCAM − phenotype resulted in established cells lines and also demonstrated a high-level invasive capability in the brains and lungs of nude mice [37].

The removal of the requirement that a CTC must express EpCAM but still be CD45− significantly increases the number of potential cells that can be called CTCs. This phenomenon is clearly seen in the dot plot of the CD45− EpCAM versus CK data for one of the patients (Figure 2b, second row, first plot). In that plot, though there are a few events in the EpCAM + CK + quadrant a much larger number of cells are present in the EpCAM − CK + quadrant. These EpCAM − CK + cell concentrations are higher in the patient samples than in those of the healthy donors (Table 2) and are associated with worse OS based on our preliminary results (Figure 4). Most striking is that in this feasibility study, with a limited number of patients, elevated levels of EpCAM − CK + CD45− cells correlated with poor OS, not just in TNBC patients but also in ER + patients. A larger pool of candidate atypical circulating cells would expand the possibility of further molecular profiling and analysis of targeted therapies. Currently, narrowly defined, traditional CTC populations can be very low in a large number of patients with metastatic breast cancer.

The confocal images presented herein confirm the heterogeneity of the populations observed in the FCM analysis with respect to CK, CD45 and EpCAM expression, including the classically defined CTC (CD45 − CK + EpCAM+), as well as of other atypical populations, including CD45 − CK + EpCAM − and CD45 − CK − EpCAM+. The size of many of these cells is similar to the size of typical white blood cells. Although it would be highly desirable to obtain estimates of the number of these different subtypes per milliliter of patient blood sample volume, such counting using a confocal microscope is not practical. It is possible to obtain cell counts using a fluorescence microscope, this technology does not have the ability to reliably distinguish between positive and negative signals at the higher wavelengths. The CM analysis also demonstrates the presence of cells that express vimentin and EGFR, possibly suggesting that these cells have undergone EMT associated with the basal subtype.

Our study findings raise several questions. Do EpCAM − CK + cells correspond to a subpopulation of CTCs with more aggressive features? These EpCAM cells with low expression are of interest because they can express mesenchymal markers such as EGFR and vimentin, biomarkers that are associated with EMT. As shown in Figures 7 and 8, CK + cells that were positive for vimentin and or EGFR were identified by ICC and FCM, and we found similar rare cells with mesenchymal markers in the blood of patients with head and neck malignancies [10]. In addition, other unusual cells were identified, including those that were negative for CD45, EpCAM and cytokeratins CK8, CK18 and CK19 but positive for vimentin, which is suggestive of the presence of an EMT phenotype. Vimentin can be present in normal blood cells. However, in all of our previous studies and the present study, we have not observed any cells that stained positive with both CD45 and vimentin, as shown in Figure 5b. Further, we have not observed any vimentin-positive cells in the blood of our healthy volunteer controls. When we visualized vimentin-positive patient samples, as shown in Figures 7 and 8, we found that the cells were either CD45− negative or positive for several other markers not associated with normal blood (CK and EGFR). These rare cell populations would not have been identified by utilizing a positive selection methodology initially targeting EpCAM.

In addition to EpCAM + and EpCAM − cell populations, herein we show that there are other abnormal circulating cell types, including the perplexing population of cells that expresses both CD45 and CK. This population has been noted by others but has not been described in detail and is believed to be primarily an artefact [1,24]. Given the limitations of the enrichment strategy, isolation technology, ICC using fluorescent dyes and the belief that the cells are artefacts, these cells have rarely been isolated and characterized.

The significant number of CD45 + CK + cells in all patient samples raises the question whether these are analysis artefacts, such as cell doublets or cells having nonspecific binding of the anti-CK antibody or the anti-CD45 antibody. In patient samples in which we used the viability dye and side scatter width settings, which is reported to exclude dead cells and obvious doublets during FCM analysis, we still observed CD45 + CK + cells. In fact, the addition of these FCM selection criteria did not significantly lower the number of CD45 + CK + events.

These data, along with multiparametric confocal images, suggest that there is a population of atypical cells that have both hematopoietic and epithelial-like characteristics that merit further investigation. Although the staining of CD68 began late in our present study, we observed these CD68 subpopulations in multiple independent patient samples. Further, this specific combination of staining is not observed in normal patient samples.

Although we acknowledge that nonspecific binding is a possible explanation for this population, the positive CD68 expression is consistently present in higher levels in metastatic patients and there is a trend toward increased levels at the time of progression. Recent reports have highlighted that increased tumor-associated macrophages in primary breast tumors are associated with worse outcomes and chemotherapy resistance [38]. However, concurrent changes in the blood have not been reported yet. Additional studies aimed at multiparametric characterization of this population, including CD16, CD14 and colony-stimulating factor receptor type 1 staining, are underway.

This study has several limitations, including a limited sample size and lack of a test and validation set to confirm correlations with clinical outcomes. However, this pilot study highlights the importance of the unbiased characterization of the atypical circulating cells that are present in metastatic breast cancer patients and that the traditional CTCs make up a minority of the abnormal circulating cells in blood samples from patients with metastatic breast cancer.

## Conclusions

To the best of our knowledge, this report is the first to describe the presence of circulating, nucleated EpCAM + CK + and EpCAM − CK + in the blood samples of a series comprising only metastatic breast cancer patients, as demonstrated by multiparametric FCM concurrently with ICC. Characterization of these subpopulations through advancements in multiparametric spectral analysis and molecular marker analysis may further elucidate the nature of these cells. Additional studies are needed to discern which of these subpopulations are most clinically relevant, although our preliminary results suggest that the CK + EpCAM − population is of interest.

## Abbreviations

CK: Cytokeratin; CTC: Circulating tumor cell; CV: Coefficient of variation; EGFR: Epidermal growth factor receptor; EMT: Epithelial–mesenchymal transition; EpCAM: Epithelial cell adhesion molecule; FCM: Flow cytometry; ICC: Immunocytochemistry; TAM: Tumor-associated macrophage; TNBC: Triple-negative breast cancer.

## Competing interests

The authors declare that they have no competing interests.

## Authors’ contributions

MBL, PB, CLS and JJC conceived of and designed the study. All authors collected and assembled the data. XP, MBL, PB, CLS and JJC analyzed and interpreted the data and wrote the manuscript. All authors read and approved the final manuscript.

## Supplementary Material

Additional file 1: Table S1 Antibodies used. 4′,6-diamidino-2-phenylindole (DAPI). Alexa Fluor (AF). Click here for file

Additional file 2 Supplemental Information. Click here for file
