# Supplementary material for: Heterogeneous atypical cell populations are present in blood of metastatic breast cancer patients
Source: Breast Cancer Res. 2014 Mar 6;16(2):R23. doi: 10.1186/bcr3622 (PMC4053256; doi:10.1186/bcr3622)
Supplement: Additional file 1: Table S1 — Antibodies used. 4′,6-diamidino-2-phenylindole (DAPI). Alexa Fluor (AF). [file bcr3622-S1.doc]

**Supplemental Table 1. Antibodies used**

| **Target** | **Antibody clone, Host Species** | **Manufacturer**  **(Catalog #)** | **Fluoroprobe** | **Secondary antibody** | **Fluoroprobe** |
| --- | --- | --- | --- | --- | --- |
| **Nucleus** | ---- | Invitrogen | DAPI | ---- | ----- |
| **Cytokeratin 8, 18, 19** | CK3-6H5, mouse | Miltenyi Biotec (130-080-101) | FITC | ---- | ----- |
| **Cytokeratin 8, 18, 19** | CK3-6H5, mouse | Miltenyi Biotec (130-090-866) | AF488,  custom conjugated | ---- | ---- |
| **Pan-CK** | Mixture Clones of C-11+ PCK-26 + CY-90 + KS-1A3 + M20 + A53-B/A2, mouse | Sigma-Alderich  (C2562) |  | anti-mouse | AF488 |
| **CD45** | HI30, mouse | BD Pharmingen (555480 ) | AF594,  custom conjugated | ---- | ---- |
| **CD68** | Y1/82A, mouse | Biolegend (333814) | PerCP-Cy5.5 | ---- | ---- |
| **EpCAM** | polyclonal, rabbit | Abcam  (ab71916) | ---- | anti-rabbit | AF647 |
| **Vimentin** | C-20, goat  or  polyclonal, goat | Santa Cruz Biotechology (SC-7557)  or  Abcam  (ab11256) | ---- | anti-goat | AF555  or  AF647 |
| **EGFR** | polyclonal, rabbit | Abcam  (ab2430) | ---- | anti-rabbit | AF647 |

**DAPI – 4',6-diamidino-2-phenylindole**

**AF – Alexa Fluor®**
