# Supplementary material for: Heterogeneous atypical cell populations are present in blood of metastatic breast cancer patients
Source: Breast Cancer Res. 2014 Mar 6;16(2):R23. doi: 10.1186/bcr3622 (PMC4053256; doi:10.1186/bcr3622)
Supplement: Additional file 2 — Supplemental Information. [file bcr3622-S2.docx]

**Supplemental Information for:**

**Heterogeneous atypical cell populations are present in blood of metastatic breast cancer patients**

Maryam B Lustberg^1,4*^, Priya Balasubramanian*^2^, Brandon Miller^2^, Alejandra Garcia-Villa^2^, Clayton Deighan, Yongqi Wu, Sarah Carothers^1,4^, Michael Berger^1,4^, Bhuvaneswari Ramaswamy^1,4^ , Erin M Olson^1,4^, Robert Wesolowski^1,4^, Rachel M Layman^1,4^, Ewa Mrozek^1,4^, Xueliang Pan^5^, Thomas A Summers^3^, Charles L. Shapiro^1,4**^, Jeffrey J. Chalmers^2, **^

^1^Stefanie Spielman Comprehensive Breast Center

Wexner Medical Center, Ohio State University (OSU)

Columbus. OH 43210

^2^William G Lowrie Department of Chemical and Biomolecular Engineering,

OSU

Columbus, OH 43210

^3^Department of Pathology and Laboratory Services

Walter Reed National Military Medical Center

Bethesda, MD 20889

^4^ The Breast Program

OSU Comprehensive Cancer Center

Columbus, OH 43210

^5^ Center for Biostatistics

The Ohio State University

Columbus, OH 43210

* Corresponding Authors with equal contributions

**Co-Senior Authors with equal contributions

**Flow cytometry spiking controls:** Ideally, one would like to set the gates for positive and negative populations based on positive and negative populations of known cell types. Consistent with this desire, a common approach is to spike a cancer cell line into normal, or in our case, buffy coat blood samples obtained from the American Red Cross. However, the significantly larger size of the cancer cell line, MCF-7 in this case, severely limits the ability of flow cytometry which is demonstrated below.

Figure 1SA is a forward scatter (FS) vs. side scatter (SS) plot of an immunomagnetically enriched population of buffy coat spiked with MCF-7 cells. In this case, we used typical detector amplification settings. Figure 1SB is a CK vs. CD45 expression plot of cells in region “B” of Figure 1SA, suggesting that the cells are blood cells. In contrast, Figure 1SC indicates that the cells in the region “C”, which includes many cells “off scale”, are MCF-7 cells.

Figure 1S


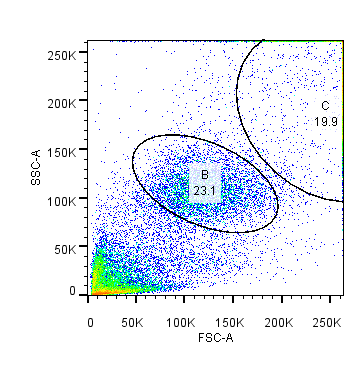

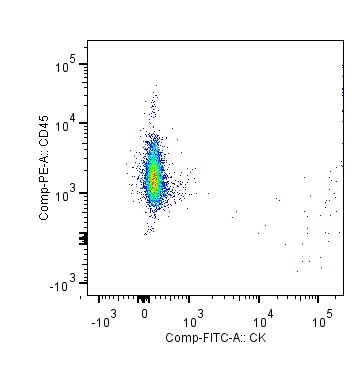

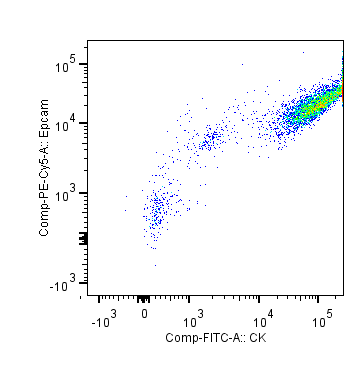


**B**

**C**

B

C

**A**

To address this “off-scale” issue, we changed the amplification to bring more of these “off-scale” cells onto the center of the FS vs. SS plot. However, as can be seen, the hematopoietic cells are “squished” into the lower left, and there are still many MCF-7 cells that are still ***off scale*** on the right hand side of Figure 2SA. Any further changes to the amplification of the signal would result in even decreased sensitivity on any “smaller” cells (we consider this current squishing of the normal cells unacceptable with respect to being able to differentiate signals. Figure 2SB and 2SC are CK vs. CD45 and CK vs. EpCAM expression for regions B and C.

Figure 2S


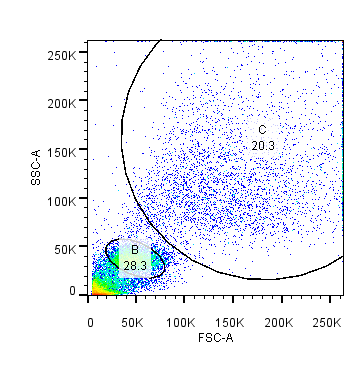

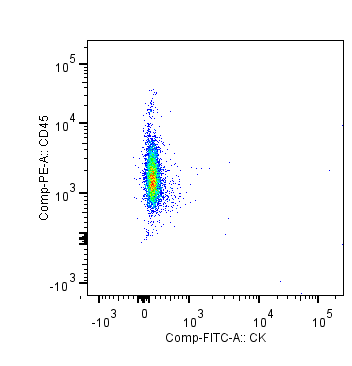

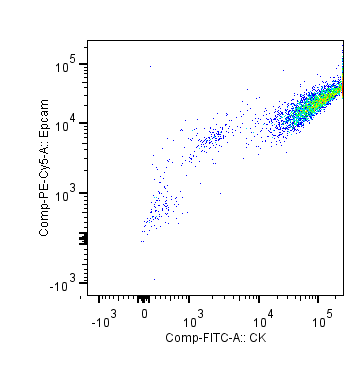


**A**

**B**

**C**

This limitation of being able to include all of the cells in the same FS vs. SS plot, and still have significant sensitivity to distinguish expression levels in the “smaller sized cells” (the squished cells in the lower left of the FS vs. SS plot), led to the discontinuation of further spiking studies.

While not extensive, a body of literature exists on the factors involved in determining the statistical significance of rare cell analysis with a flow cytometer.[[23-26](#_ENREF_23)] Issues surrounding establishment of the actual number of true events, as well as a measure of the significance of a rare events once it is detected can be categorized into at least three distinct factors: 1) artifacts that can appear as a true “event”, 2) a sufficient signal to noise ratio to allow appropriate setting of “gates” to determine a positive from a negative event, and 3) the frequency of the population of interest which has been shown to typically follow a Poison distribution. With respect to issue 1, it is well known that cell debris and dead cells can bind antibodies. In our study we initially gated the forward and side scatter to remove events on the outer edges of the plots which are known, based on operator experience in our Analytical Cytometry Core, to contain events that are not cells. To assist in setting the gates, we used the fluorescence minus one approach for each laser to determine the fluorescence intensity distribution of unstained cells. However, when cells are weakly expressing a surface marker, this distinction is less clear. The dot plots of SS versus CD45 expression for the patient (top row, third plot, Figure 2) demonstrates this challenge in setting gates for positive and negative events: the line separating the CD45- from CD45+ weak divides a continuous population of cells. This type of condition was even more prevalent, for more than just CD45 expression, in other patient samples.

From equation 1, the larger the number of positive numbers observed corresponds to a smaller value of CV, which is equivalent to a more reliable measurement. To achieve a reasonably accurate measure of a CV of 5% requires 400 total events to be positively detected. As expected, the amount of traditional CTC (CD45-CK+EpCAM+) in the patient samples are very small; below our requirement of a CV of 5%. The small number of CTCs are not only hard to be detected using FCM, but also difficult to be used for clinical association analysis due to the large CVs (less accurate measure). However, EpCAM-CK+CD45- events are present in much higher numbers than CD45-CK+EpCAM+ events in the patient samples; consequently, we can easily obtain a CV of 5% during FCM analysis which would make this methodology much more practical to utilize for clinical samples.

Epifluorescent and Confocal Images Figure S3A, B and C presents epifluorescent images of normal blood, MCF-7 and MDA-231 spiked into normal blood cells, and stained for: DAPI, anti- pan-CK-AF488, anti-CD45-AF594, and anti-EpCAM-AF647, respectively.

**Figure S3A: Normal blood cells**


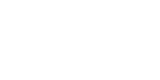

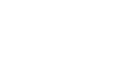

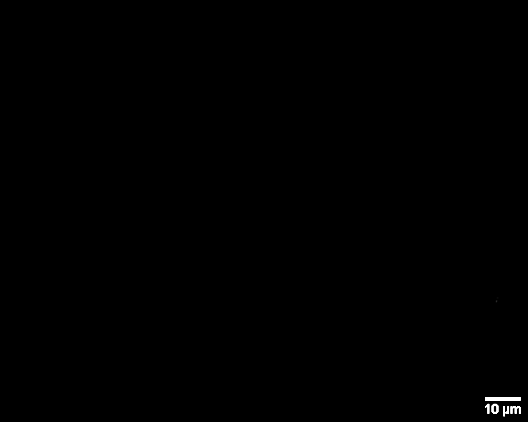

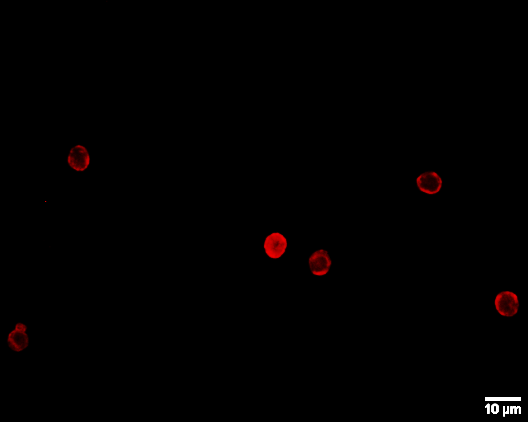

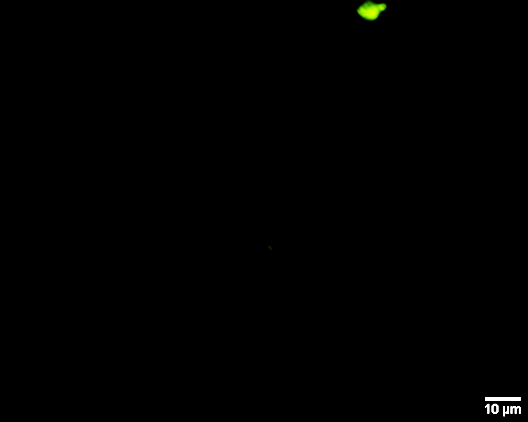

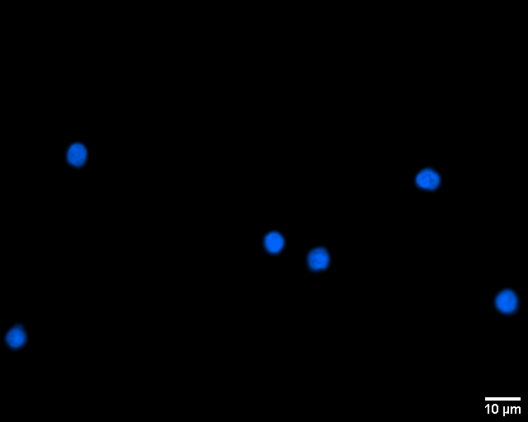


CD45

CK

**Figure S3B: MCF-7 + blood cells**


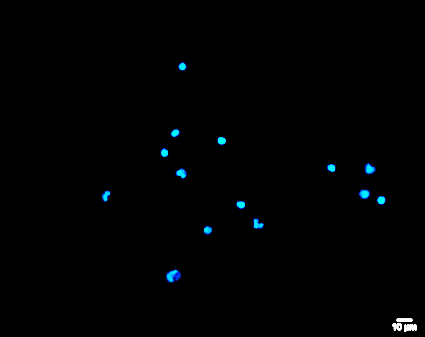

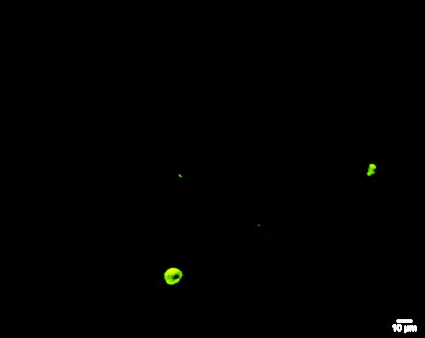

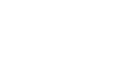


CK


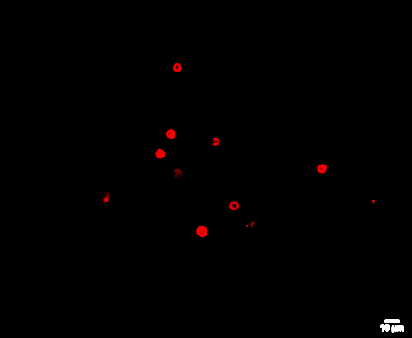

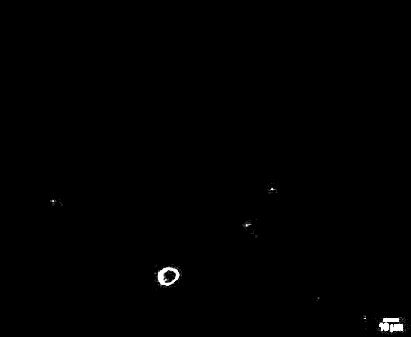

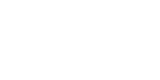


CD45

**Figure S3C: MDA-MB-231 + blood cells**


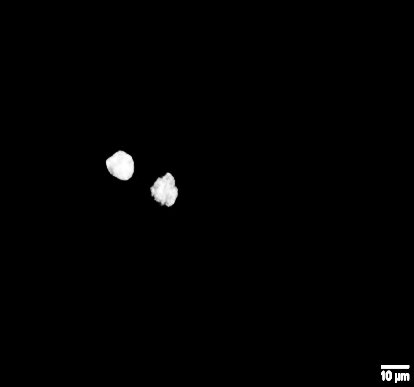

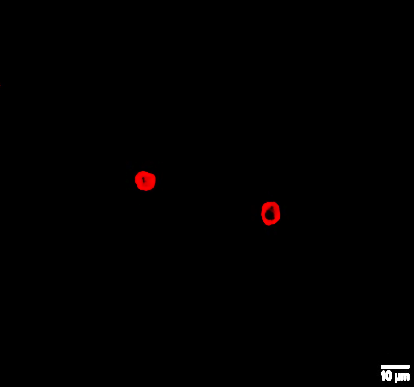

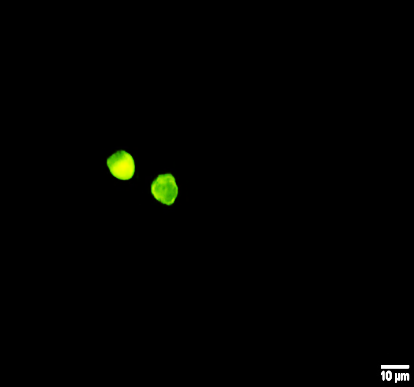

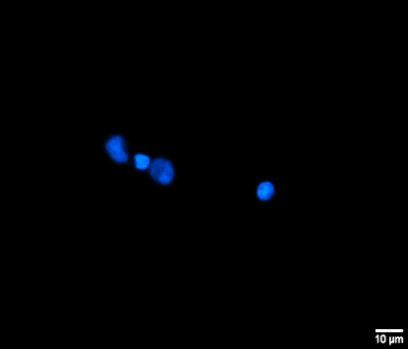


DAPI

EpCAM

CD45

CK

Figure S4 and Figure S5 present the emission and excitation spectra, as well as the excitation and emission filters used in this study for the epifluorescent studies as well as the laser lines and emission filters used for the confocal studies. Note the significant decrease in spectral overlap, especially with respect to dye excitation, when laser excitation is used on the confocal system.

**Figure S4**


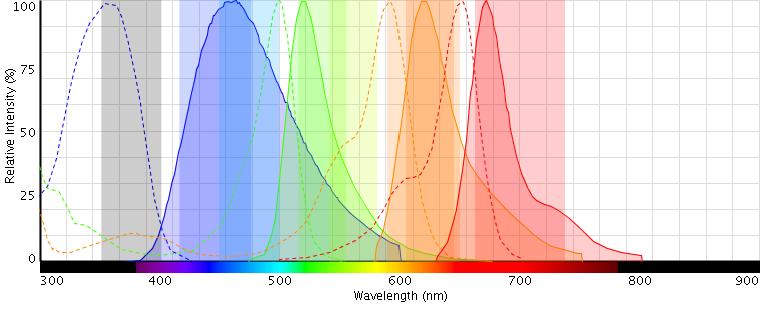


Excitation

Emission

DAPI:

AF488:

AF594:

AF647:

**AF488**

**filters**

**AF594**

**filters**

**AF647**

**filters**

**DAPI**

**filters**

**Figure S5**

Laser lines


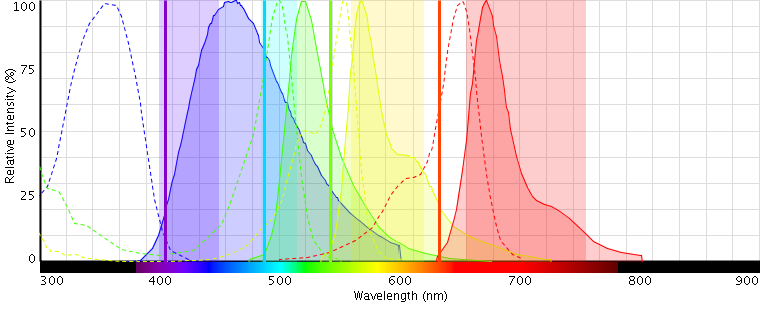


Figures S6A and S6B are representative, epifluorescent images of a four color stained cytopsins of a patient blood samples prior to (S6A), and after (S6B), magnetically enriched blood.

**Figure S6A: Prior to magnetic enrichment**


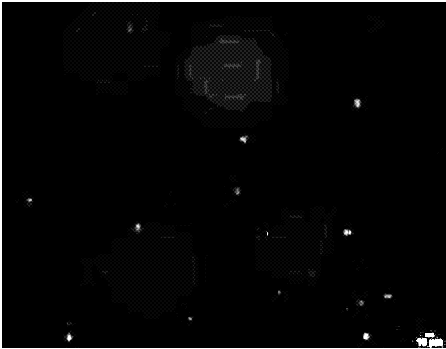

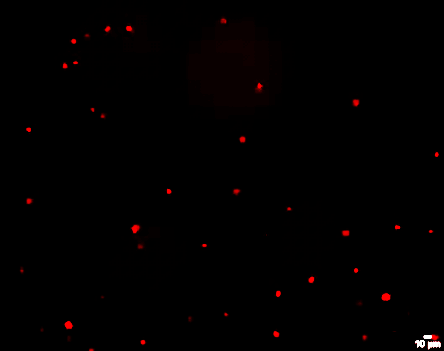

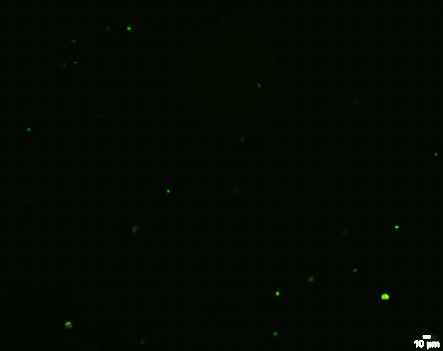

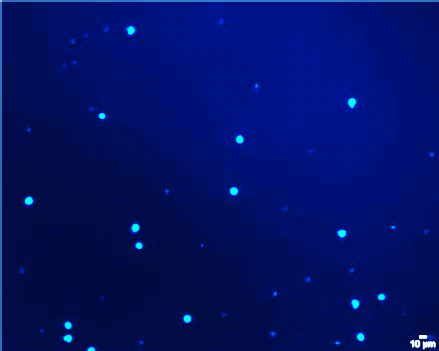


EpCAM

CD45

CK

DAPI

**Figure S6B: After magnetic enrichment**


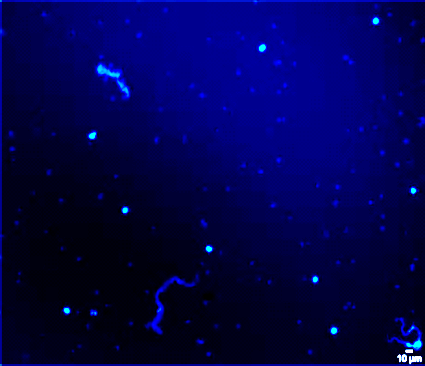

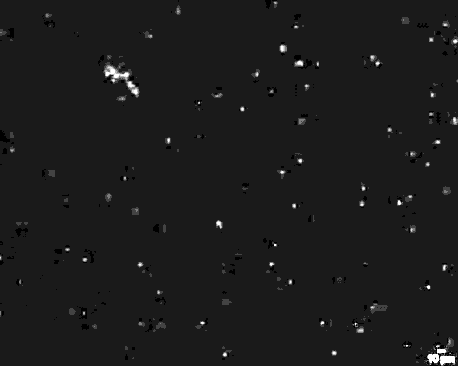

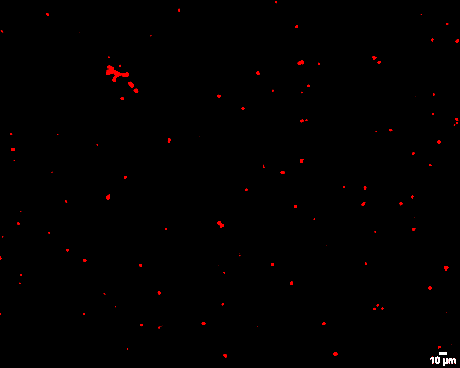

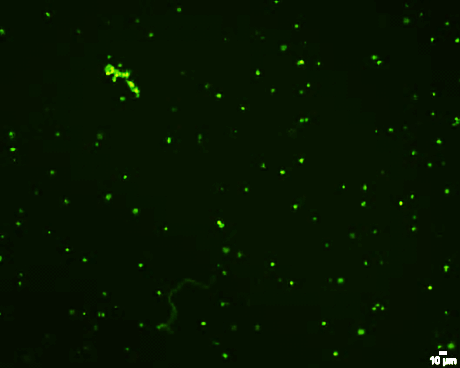


CD45

EpCAM

CK

DAPI

**Isotype Controls**

Three isotype antibody controls were tested: 1) Mouse IgG1,κ, FITC conjugated (BD Pharmingen, Clone: MOPC-21, Cat:555748), 2) Mouse IgG1,κ, PE conjugated (BD Pharmingen, Clone: MOPC-21, Cat:555749), and 3) Rabbit polyclonal IgG, FITC conjugated. For all of the isotypes tested, the fluorescent intensity was nearly the same as the unstained control. An example for one of these controls is presented in Figure S7.

**Figure S7**


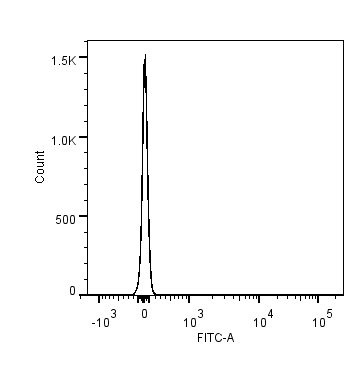

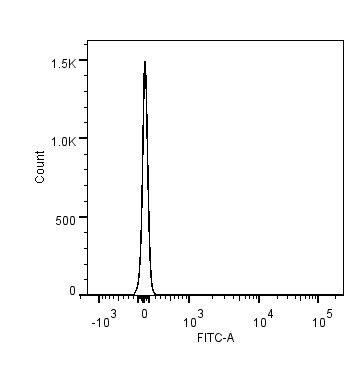

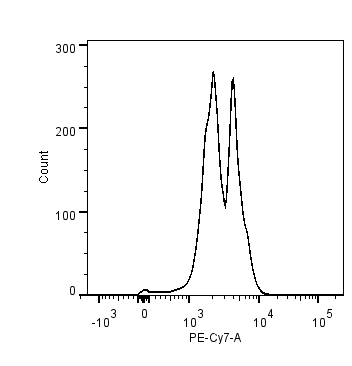


**Unstained**

**Stained with CD45-PE/Cy7**

**Stained with Isotype-FITC**

Stained with Isotype-FITC

Stained with CD45-PE/Cy7

Unstained
